# Multimorbidity and dementia risk in hospitalised patients: an administrative data linkage study of hospital patients in Northern Ireland.

**DOI:** 10.23889/ijpds.v7i3.2012

**Published:** 2022-08-25

**Authors:** Emma Curran, Michael Rosato, Gerard Leavey

**Affiliations:** 1 Ulster University

## Introduction

While chronic disease is a risk factor for dementia (Shang et al., 2020) studies addressing multimorbidity and dementia risk are limited. Multimorbidity leads to steeper dementia progression (Bauer, 2014; Haaksma, 2019) impacting cognitive dysfunction, cognitive decline and impairment (Wei, 2020; Vassilaki, 2015). Multimorbidity has generally been examined using a dose- response disease count, something cited as impractical for clinicians due to information deficits on identifying disease specific pathways (Whitty, 2020). Thus, differentiating disease clusters and dementia risk will provide insight into the pathophysiology of dementia, while informing priority targets for early intervention, potentially reducing burden and secondary care costs.

## Aim

The COVID-19 pandemic has resulted in delayed diagnosis and treatment for cancer patients and increases in elective surgery waiting lists. The impact on other ‘long-term’ conditions (LTCs) is unclear. We examined the effects of the pandemic on the recorded incidence of 20 LTCs to inform decisions on treatment pathways and resource allocation.

## Data linkage and analysis

We used national data defined by the NHAIS, including basic demographics for all patients aged fifty-five plus and registered with a GP in Northern Ireland. The linked data comprised: Patient Admissions and Discharges (from 2013-2021); Electronically captured prescribing data, area-type of usual residence; Multiple Deprivation Indicators; and GRO mortality data (including cause of death). We identified multimorbidity through validated algorithms for ICD-10 coding of thirty morbidities in administrative hospital diagnostics data (Tonelli et al., 2019). The same data identified dementia-free patients at baseline. Latent Class Analysis assigned patient groups by the patterning of multimorbidity states, following them through the study period, with analysis in relation to subsequent diagnoses of both clinical dementia and mortality. As age has a potential modifier effect we tested through age group stratified analyses. A series of multinomial logistic regression models further examine (for the multimorbidity sub-groups of interest) risks associated socio-demographic, socio-economic, medication use and household indicators at the individual, family and area level.

## Results

Multimorbidity occurs in two thirds, approximately, of this hospital population of older adults. By age sixty-five years, most people are multimorbid. We identify subgroups of multimorbidity associated with dementia onset and mortality, combinations of diabetes, hypertension, chronic kidney disease and cancer multimorbidity are highlighted as higher-risk conditions for increased dementia incidence and mortality. Greater disease burden is associated with frequent healthcare service use, medication use and is more common in the most socioeconomically deprived, compared with the least deprived.

## Discussion

Evidence on multimorbidity patterns and dementia is limited (Grande, 2021). Our study suggests that people with combinations of diabetes, hypertension, chronic kidney disease and cancer multimorbidity are associated with increased risks for dementia and socio demographic and economic circumstances further increase this risk. Overlapping combinations comprising chronic kidney disease + hypertension, or diabetes + hypertension, were a significant proportion of total secondary care costs for patients with multimorbidity; combinations comprising chronic heart failure + chronic kidney disease + hypertension had the highest proportion of total cost from potentially preventable emergency admissions (Stokes et al., 2021). Our finding support the body and mind connection in dementia development. Thus, we note that cardiovascular health is associated with dementia (Grande, 2021). Often described as a heart-brain connection, cardiovascular disease leads to higher dementia risk due to a number of mechanisms including but not limited to chronic inflammation (Grande, 2021). Identifying such high-risk groups will facilitate tailored interventions for dementia prevention and the reduction of healthcare use and costs.

